# Prediction of thrombotic events in patients with autoimmune hemolytic anemia: a multicenter retrospective observational study

**DOI:** 10.1007/s11239-025-03129-8

**Published:** 2025-06-22

**Authors:** Lucie Carneiro Esteves, Lucile Grange, Jean-Baptiste Gaultier, Baptiste Gramont, Emilie Chalayer, Martin Killian

**Affiliations:** 1 Department of Internal Medicine, CHU de Saint-Etienne, Saint-Etienne Cedex 02, France; 2 CIRI - Centre International de Recherche en Infectiologie, Team GIMAP, Université de Lyon, Université Jean Monnet, Université, Claude Bernard Lyon 1, INSERM, U1111, CNRS, UMR530, F42023, Saint-Etienne, France; 3 Clinical Investigation Center, INSERM CIC-EC 1408, Saint-Etienne, France; 4 SAINBIOSE, UMR 1059, INSERM, Université Jean Monnet, Saint- Etienne, France; 5 Department of Hematology and Cell Therapy, Institut de Cancérologie Universitaire, CHU de Saint-Etienne, Saint-Etienne, France

**Keywords:** Autoimmune hemolytic anemia, Venous thromboembolism, Arterial thromboembolism, Thrombotic events

## Abstract

**Background:**

Autoimmune hemolytic anemia (AIHA) is recognized to increase the risk of thrombotic events (TE), including venous thromboembolism (VTE) and arterial thromboembolism (ATE), but little is known about specific risk factors and characteristics.

**Methods:**

This retrospective multicenter observational study, sought to assess TE incidence, identify associated thrombotic risk factors and assess the external validity of the Padua score in AIHA for predicting VTE.

**Results:**

TE incidence during the study period was 25% (CI95%: 17–36), consisting of 19 VTE in 16 patients (18% [CI95%: 9–28]) and 11 ATE in 7 (8% [CI95%: 4–16]). A high number (≥ 5) of hemolysis attacks was associated with overall TE (OR 6.9 [CI95%: 1–82], *p* = 0.03). Univariate analysis confirmed splenectomy and VTE history as the strongest VTE-related risk factors (OR 7.5 [CI95%: 1–44], *p* = 0.009 and OR 3.8 [CI95%: 1–14], *p* = 0.04), whereas having primary warm AIHA was identified as a novel risk factor (3.1 [1–11] *p* = 0.05) which needs to be confirmed in further studies. ATE risk factors were age**≥** 80 years at diagnosis (OR 8.9 [CI95%: 1–68] *p* = 0.02), and having ≥ 3 cardiovascular risk factors (OR 8.9 [CI95%: 1–70] *p* = 0.01). The area under the Receiver Operating Characteristic curve of the Padua score was 0.66.

**Conclusions:**

TE incidence is high in AIHA, especially when there are repeated hemolysis attacks and associated VTE and/or ATE-related risk factors, thus warranting the conduct of prospective clinical trials to allow for an improved TE risk stratification and to design adapted management for both VTE and ATE.

**Graphical Abstract:**

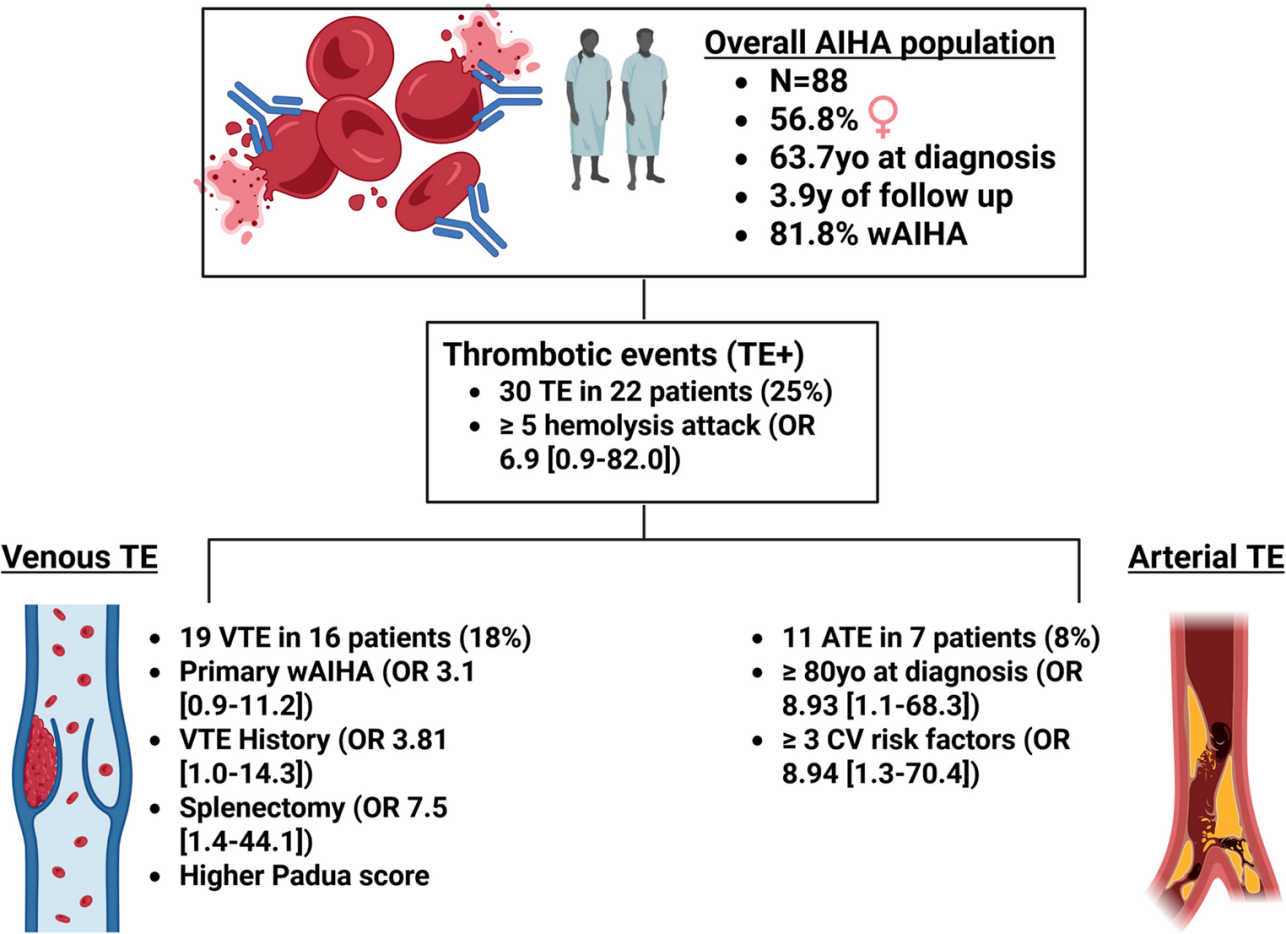

**Supplementary Information:**

The online version contains supplementary material available at 10.1007/s11239-025-03129-8.

## Key points

- One quarter of the AIHA patients presented thrombotic events (TE) in our cohort.
- Venous TE (VTE) was associated with primary wAIHA, splenectomy and VTE history.
- The Padua score was higher in VTE + and could help identify patients at higher risk.
- Arterial TE (ATE) was associated with age ≥ 80 and high CV risk.
- Further prospective TE-dedicated studies are warranted in AIHA.

**Supplementary Information:**

The online version contains supplementary material available at 10.1007/s11239-025-03129-8.

## Introduction

Autoimmune hemolytic anemia (AIHA) is characterized by enhanced hemolysis induced by autoantibodies directed against the erythrocyte membrane, with or without complement activation [1, 2]. The warm antibodies associated with AIHA (wAIHA) usually consist of IgG alone or associated with the complement C3d, with the highest antigen affinity at 37 °C. It accounts for 48 to 70% of AIHA cases [2]. The cold antibodies associated with AIHA (cAIHA) usually of the IgM isotype, optimally binding to RBCs at 0–4 °C, are involved in 15–25% of AIHA cases. AIHA is typically classified as primary if not associated with any other pathologies, and as secondary if associated with underlying diseases, including systemic lupus erythematosus (SLE), hematologic neoplasms, or autoimmune conditions [3]. This rare disease, whose diagnosis is based on the direct antiglobulin test (DAT; also called Coombs test) [4], has an estimated incidence of 0.5-1 cases per 100,000 per year [5] and can be life-threatening, with a mortality rate of 8–15% [6, 7].

In few retrospective and case control studies, AIHA was linked to an increased risk of venous thromboembolism (VTE) [8–11]. Hemolysis itself can now be considered as a recognized risk factor for VTE [7–9, 12–17], whereas evidences are scarce for the antiphospholipid syndrome (aPLs) playing a significant risk factor for thrombotic events (TE) in AIHA [18]. However, there are still multiple unknowns and unmet needs in AIHA regarding the thrombotic risk. So far, the guidelines about AIHA treatment do not recommend any thromboprophylaxis except for an extended period of postoperative thromboprophylaxis [2]. Moreover, the Padua prediction score was calculated on an AIHA cohort but the choosing time at score calculation was not the same for patients with thrombosis and without thrombosis [13]. No study has assessed the thrombotic risk in AIHA depending on their subtypes. Finally, little is known about AIHA-associated arterial thrombotic events (ATE) (acute myocardial infarction [AMI], ischemic stroke).

The identification of specific risk factors in AIHA is paramount to enhance patients’ management and survival. This study sought to assess the frequencies of venous and arterial TE in AIHA patients, identify the associated risk factors, and assess the external validity of the Padua score to predict VTE.

## Materials and methods

### Study design and population

This multicenter retrospective study was conducted at 3 centers: Saint-Etienne University Hospital, Lucien Neuwirth Cancer Center and the “Etablissement Français du Sang” (French Blood Establishment; EFS) site in Saint-Etienne. Included patients were diagnosed between January 1, 2010 and December 31, 2018, and followed up until April 30, 2020.

Three databases were analyzed for patients’ recruitment. For hospitalized patients, data were collected via each institution’s data on hospital stays i.e., the French “Programme de Médicalisation des Systèmes d’Information” (PMSI). Patients receiving outpatient care were identified based on DAT results ≥ 2, extracted from EFS’ laboratory database, before accessing the patients’ medical files.

Inclusion criteria were as follows: ≥18-year-old patients at inclusion with AIHA diagnosis according to international guidelines [2]. Patients with Evans syndrome were included if they met these criteria. Patients with other causes of hemolytic anemia (constitutional, mechanical, toxic, or bacterial), those with DAT-negative hemolytic anemia, were not included. The study was approved by Saint-Etienne University Hospital Ethics committee (“Terre d’Ethique”; IRBN752020/CHUSTE).

### Study objectives and data collection

The primary goal of this study was to assess the incidence of TE in AIHA patients. TE were identified through imaging confirmation of conditions such as deep-vein thrombosis, central venous catheter-related thrombosis, pulmonary embolism, or any acute cardiovascular incidents (e.g., AMI, ischemic stroke, peripheral arterial thrombosis, and other less common occurrences like atrial thrombosis), as well as sudden, unexplained deaths (presumed caused by pulmonary embolism, AMI, or stroke). Moreover, to identify factors associated with TE, data were collected from patients’ files, including: gender, age at AIHA diagnosis and at inclusion, co-morbidities, VTE and cardiovascular risk factors, previous VTE history, splenectomy, Padua score items [19], and anticoagulant treatments, biological including DAT results, as well as clinical and morphological examination during the AIHA attacks, hospitalizations, AIHA treatments, TE (VTE or ATE) occurrence and treatments implemented, short-term survival at 1 year, and overall survival, date of last contact in case of lost to follow up, and cause of death if applicable.

AIHA was considered primary if no associated cause was identified, including neoplasia (solid or hematologic, especially lymphoproliferative neoplasms), autoimmune diseases, infectious diseases, post-allogeneic transplantation, or drug intake identified as causal. All other cases were considered secondary AIHA. Based on the DAT results, the following distinction was made: wAIHA (IgG alone or associated with C3d), cAIHA (C3d alone associated with high cold agglutinin titer ≥ 64), or mixed AIHA (IgG associated with C3d and high cold agglutinin titer ≥ 64). In cases of inconsistency between DAT results and clinical context, the opinion of the patient’s attending physician was sought after.

### Statistical analysis

Descriptive statistics were used to summarize patients’ clinical characteristics. Quantitative data were represented as means or medians with minimum/maximum or IQR values. Qualitative data were shown as counts and percentages. We defined baseline as AIHA diagnosis. Univariate comparisons using Fisher’s exact test or chi-square or Student’s t-tests were used to identify risk factors associated with TE, and when significantly different, their Odds Ratio and 95% confidence interval were calculated. The subgroup analysis was intended for VTE and ATE. Due to the limited number of events, we did not perform a multivariate analysis, as it would not have been statistically reliable [20]. For the Padua score, comparisons between groups were conducted only if every data was available. We assessed the Padua score discrimination by calculating the Receiver Operating Characteristic (ROC) curve. Statistical significance was set at *p* ≤ 0.05. The statistical analysis was performed using R software.

## Results

### General characteristics

**Fig. 1:**
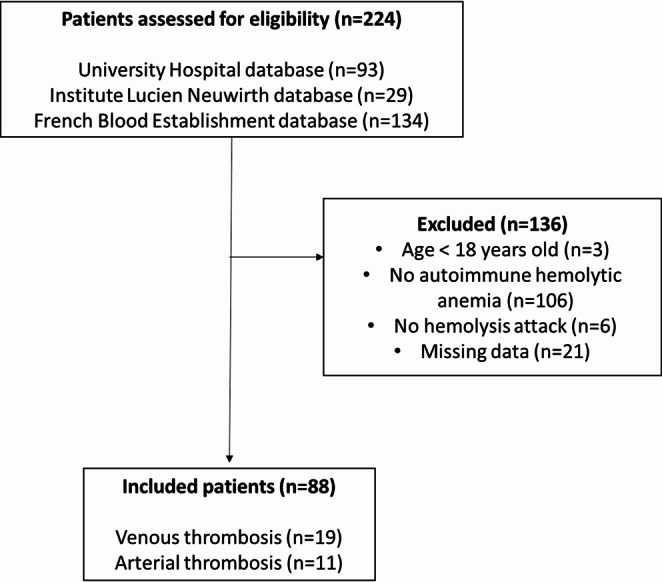
Study flowchart

Overall, 224 medical files from the three databases were analyzed. One hundred and thirty-six patients were excluded, mainly due to their anemia being not recognized as AIHA by the attending physician (*n* = 83, 61%). The study flowchart is detailed in Fig. 1.

Baseline characteristics of patients are presented in Table 1. Among the 88 included patients, the patients’ median age was 67.5 (8–91) years at initial AIHA diagnosis. The mean follow-up duration was 3.9 years (0-9.8). The mean age at death was significantly higher in the TE + group (83 vs. 74 years, *p* = 0.001). No difference was found between the TE subgroups in terms of overall survival duration. The main cause of death was infections (*n* = 10, 31.3%). Interestingly, 3 (9.4%) TE-related deaths were reported in the whole cohort, and none due to hemorrhagic complications. Data are summarized in Table 1.

No patients received long-term thromboprophylaxis. Regarding patients receiving therapeutic anticoagulation, 3 had atrial fibrillation, 3 has previous VTE and one had a mechanic valve. Patients received anti vitamin K treatment or heparins. Only one patient received direct oral anticoagulant.

**Table 1:** Characteristics of AIHA patients at inclusion

			Total (*n* = 88)	AIHA TE- (*n* = 66)	AIHA TE+ (*n* = 22)
Women, n (%)			50 (56.8%)	35 (53%)	15 (68.2%)
Follow-up	Mean duration (days)		1,429	1,355	1,652
Mean age upon diagnosis (years)			63.7	63.1	65.7
wAIHA			**72 (81.8%)**	**53 (80.3%)**	**19 (86.4%)**
	Primary, n (%)		30 (41.7%)	19 (35.8%)	11 (57.9%)
	Secondary, n (%)		42 (58.3%)	34 (64.2%)	8 (42.1%)
	SLE, n (%)		6 (14.3%)	6 (17.6%)	0 (0%)
	Hematologic neoplasm, n (%)		32 (76.2%)	26 (39.4%)	6 (27.3%)
	Others, n (%)		4 (9.5%)	2 (3%)	2 (9.1%)
	Evans syndrome, n (%)		13 (14.8%)	8 (12.1%)	5 (22.7%)
cAIHA			**16 (18.2%)**	**13 (19.7%)**	**3 (13.6%)**
	Primary, n (%)		7 (53.8%)	6 (60%)	1 (33.3%)
	Secondary, n (%)		6 (46.2%)	4 (40%)	2 (66.7%)
	CAD, n (%)		13 (81.3%)	10 (76.9%)	3 (100%)
	Post-infection, n (%)		3 (18.7%)	3 (23.1%)	0 (0%)
Comorbidities	Mean number of CV risk factor per patient (n)		2.1	2.02	2.4
	Splenectomy, n (%)		9 (10.2%)	4 (6%)	5 (22.7%)
	aPLs				
	- Testing, n (%)		32 (36.4%)	22 (33.3%)	10 (45.5%)
	- Positive, n (%)		11 (34.4%)	9 (40.9%)	2 (20%)
TE risk factors	VTE history, n (%)		19 (21.6%)	11 (16.7%)	8 (36.4%)
	Hospitalization, n (%)		65 (73.9%)	47 (71.2%)	18 (81.8%)
Disease characteristics	Symptomatic hemolysis, n (%) Hemolysis attacks, n (%) - 1–2 - 3–4 - ≥ 5		77 (87.5%) 71 (80.7%) 11 (12.5%) 6 (6.8%)	57 (86.4%) 57 (86.3%) 7 (10.6%) 2 (3%)	20 (90.9%) 14 (63.7%) 4 (18.1%) 4 (18.2%)
	**Biological parameters**, mean				
	Hb (g/dL)		7.99	7.95	8.14
	LDH (UI/L)*		1,021	1,067	854
	Free bilirubin (µmol/L)*		34	37	26
Antithrombotic treatment at AIHA diagnosis					
	APT, n (%)		18 (20.5%)	14 (21.2%)	4 (18.2%)
	DAPT, n (%)		2 (2.3%)	1 (1.5%)	1 (4.5%)
	Therapeutic-intensity anticoagulation, n (%)		7 (8%)	4 (6.1%)	3 (13.6%)
	Prophylactic-intensity anticoagulation, n (%)		0 (0%)	0 (0%)	0 (0%)
Mortality					
1-year status	Survival, n (%)		77 (87.5%)	59 (89.4%)	18 (81.8%)
	Death, n (%)		6 (6.8%)	4 (6.1%)	2 (9.1%)
	Unknown, n (%)		5 (5.7%)	3 (4.5%)	2 (9.1%)
Mean age at end of follow-up (years)			68.8	67.6	72.4
End of follow-up status	Survival, n (%)		51 (58%)	40 (60.6%)	10 (45.5%)
	Death, n (%)		32 (35.2%)	23 (34.9%)	9 (40.9%)
	Unknown, n (%)		5 (5.7%)	3 (4.5%)	3 (13.6%)
Mean age at death (years)			76.4	73.8	83.1
Cause of death	Infection, n (%)		10 (31.3%)	9 (39.1%)	1 (11.1%)
	Cancer, n (%)		3 (9.4%)	3 (13%)	0 (0%)
	Hematologic neoplasm, n (%)		4 (12.5%)	3 (13%)	1 (11.1%)
	Cancer + Hematologic neoplasm, n (%)		1 (3.1%)	1 (4.3%)	0 (0%)
	TE, n (%)		3 (9.4%)	-	3 (33.3%)
	AIHA, n (%)		1 (3.1%)	1 (4.3%)	0 (0%)
	Other, n (%)		4 (12.5%)	3 (13%)	1 (11.1%)
	Unknown, n (%)		6 (18.8%)	3 (13%)	3 (33.3%)

AIHA: autoimmune hemolytic anemia; CI confidence interval, CAD: cold agglutinin disease; cAIHA: cold AIHA; CV: cardiovascular; Hb: hemoglobin; aPLs: anti-phospholipid antibody syndrome; APT: antiplatelet therapy; DAPT: dual antiplatelet therapy; LDH: lactate dehydrogenase; CRP: C-reactive protein; OR: odds ratio, SLE: systemic lupus erythematosus; TE: thrombotic event; VTE: venous thromboembolism. wAIHA: warm AIHA; *: Presence of missing data

### Thrombotic events incidences

During follow-up, 30 TE were recorded in 22 patients (25% [CI95%: 17–36]), consisting of 19 VTE in 16 patients (18% [CI95%: 9–28]) and 11 ATE in 7 (8% [CI95%: 4–16]), including one who had experienced one VTE and several ATE episodes. Twenty-three (26% [18–37]) patients had never been hospitalized for their AIHA, including two patients who exhibited VTE episode and two others with ATE.

Regarding univariate analysis, significant results are presented in Table 2; the presence of ≥ 5 hemolytic attacks being the only factor associated with overall TE. APLs was sought after more frequently in TE + cases, though its actual presence did not differ between groups. C-reactive protein (CRP) levels and concomitant infection did not differ between groups.

**Table 2:** Univariate analysis of risk factors associated with thrombotic events

	OR (CI 95%)	*p*
*General TE risk factors*		
≥5 hemolysis Attacks, n (%)	6.90 (0.9–82.0)	0.032
*VTE risk factors*	***OR (CI 95%)***	***p***
Primary wAIHA, n (%)	3.1 (0.9–11.2)	0.047
VTE history, n (%)	3.81 (1.0-14.3)	0.038
Splenectomy, n (%)	7.50 (1.4–44.1)	0.009
*ATE risk factors*	***OR (CI 95%)***	***p***
Age ≥ 80 years at diagnosis, n (%)	8.93 (1.1–68.3)	0.022
≥ 3 CV risk factors, n (%)	8.94 (1.3–70.4)	0.012

ATE: arterial thromboembolism; CI confidence interval, CV: cardiovascular; OR: odds ratio, TE: thrombotic event; VTE: venous thromboembolism

### Venous thrombosis risk

Patient VTE + and VTE- subgroups were compared (Supplementary Table 1), and significant data regarding VTE-associated risk factors (splenectomy, previous VTE, wAIHA) were summarized in Table 2. VTE diagnosis was made at a median of 15 (0-115) days post-AIHA onset. VTE occurred mainly during the first AIHA attack (*n* = 10; 62.5%). In almost half of the cases (*n* = 7, 44%), no specific treatment for AIHA was in place at the time of VTE diagnosis. Patients with treatments received corticosteroid therapy, either alone (*n* = 6), combined with chemotherapy (*n* = 1) or concomitant splenectomy (*n* = 1). All patients survived this first VTE episode; one patient died because of the thrombotic episode after the second AIHA attack.

### Arterial thrombosis risk

Considering the seven patients displaying 11 ATE events, 4 (57%) presented ischemic strokes, and 3 (43%) AMI. No AIHA treatment was in place for 3 patients (42.9%), while the other 4 were on corticosteroid therapy, alone or in combination. A second ATE episode occurred in four patients (57.1%), two (18.1%) suffering from ischemic stroke and the remaining two (18.1%) from limb ischemia. Of these, one patient had no treatment for his AIHA, while the other three were on corticosteroid therapy, either alone or in combination.

Patient subgroups with arterial thrombosis (ATE+) and without (ATE-) were compared (Supplementary Table 2), with significant data (older age and high number of cardiovascular risk factors) summarized in Table 2. Among the cardiovascular risk factors, having diabetes and a personal history of AMI were significantly associated with ATE (*p* = 0.05 and *p* = 0.022, respectively).

### Padua score prediction

Using Padua score, 37 patients (42%) were classified as being at high-risk (≥ 4). Patients in this group had a cumulative incidence of VTE of 27% (95% CI: 14–44%). The score ranged from 0 to 10, with a median of 4 (IQR 3–6.2, *n* = 16) in the thrombosis group whereas the median was 3 (IQR 1–4, *n* = 72) in the group without thrombosis (*p* = 0.04).

**Fig. 2:**
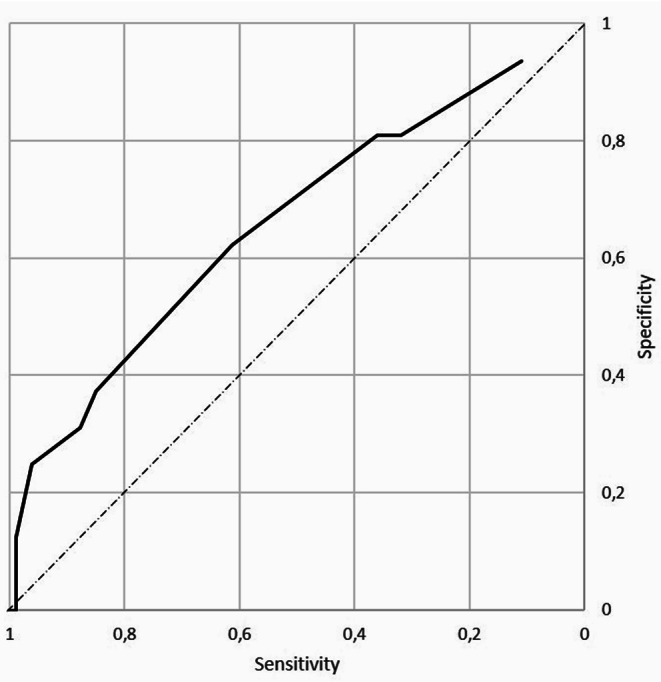
ROC IMPEDE Padua score, AUC = 0.66. ROC: receiver operating characteristic, AUC: Area under the curve

Padua items significantly associated with VTE were previous VTE (*p* = 0.01) and reduced mobility (*p* = 0.001). The area under the ROC curve (AUC) for the Padua score was 0.66 (Fig. 2).

## Discussion

The herein reported data provide further evidence supporting that patients with AIHA are at very high risk of TE complications, either arterial, venous or both. With a median follow-up of 3.9 years in our cohort, the TE incidence was 25% (CI95%: 17–36), including 18% (CI95%: 9–28) VTE and 8% (CI95%: 4–16) ATE. However, despite these high frequencies, guidelines for the prevention and management of AIHA-associated TE are still lacking, due to insufficient available evidences, except for a recommended extended postoperative thromboprophylaxis period [2]. Hence, further research are warranted.

First, the current findings are consistent with previous studies (Table 3).

Clinicians should be aware of this high risk of TE in AIHA, particularly during hemolytic phases especially when they are repeated (≥ 5 episodes during the follow-up period conferring an OR of 6.9 [95% CI: 1–82]). However, the intensity of hemolysis was not significantly associated with the risk. These observations suggest that while hemolytic activity may serve as a trigger for thrombotic events, its severity does not necessarily correlate with the risk of developing TE.

Recent surgery (< 1 month) was not significantly associated with TE risk in our cohort, probably because of the use of thromboprophylaxis. The presence of aPLs, which is often linked to thrombotic risk, did not differ significantly between the TE + and TE- groups, suggesting that other factors are at play in the increased thrombotic tendency observed in AIHA. Despite the common autoimmune mechanisms, APLs’ role has not been demonstrated to date in AIHA, except in one study where lupus anticoagulant was involved [15].

Regarding VTE risk, > 80% of VTE occurred during the first two AIHA attacks, with a short median time period (15 days) between AIHA diagnosis and VTE occurrence, and no patient who had received thromboprophylaxis. In line with these elements, prophylactic anticoagulation should be considered early in these AIHA patients. Moreover, for the first time, our study shows that this risk is significantly associated with wAIHA. This could be explained by the role of complement C3 in thrombotic risk [21]. Complement activation has been implicated in the pathogenesis of both hemolysis and thrombosis, and its role in wAIHA may contribute to the increased risk of TE in these patients. In their study, Lecouffe-Desprets et al. [9] noted that all VTE complications (8 out of 40 patients) occurred in the primary wAIHA group, while Mauro et al. [22] reported no TE in 52 patients with AIHA secondary to chronic lymphocytic leukemia (CLL). Audia et al. [8] and Roumier et al. [23] did not find this increased VTE risk in primary wAIHA patient groups in their studies, which have differences compared to ours. Study by Audia et al., did not use the same inclusion criteria (haptoglobin < 0,2 g/l) which excluded potential AIHA with concomitant inflammation versus our inclusion criteria based on international guidelines which did not exist at that time [2]. In Roumier et al., the cohort was not well balanced regarding splenectomy: 22% of the patients underwent splenectomy in the secondary wAIHA cohort versus 4% in the primary wAIHA. Thus, our finding about a possible higher VTE risk in primary wAIHA underscores the need for further research in this population, especially about the underlying mechanisms of thrombosis in AIHA. Splenectomy is significantly associated with VTE risk (OR: 7.5 [CI95%: 1.4–44.1], *p* = 0.009) in our study as previously reported (Table 3). Splenectomy has historically been associated with an increased risk of thrombosis, particularly during the immediate postoperative period. For patients who have undergone splenectomy, thrombotic prevention should be particularly considered from the time of AIHA diagnosis and after surgery. The current trend of declining splenectomy rates due to alternative therapies, such as rituximab, may contribute to a certain reduction in thrombotic complications. In our study, the two patient subgroups (VTE + and VTE-) were not different in terms of symptoms and biological parameters.

Regarding treatments, nearly half of the patients were not receiving any treatment for AIHA at the time of VTE occurrence, while one-third were on high-dose corticosteroids. Although several studies have highlighted a pro-thrombotic role of corticosteroids [24, 25], our study suggests that other pro-thrombotic factors are involved in this setting, including hemolytic flare-ups themselves which usually benefit from high-dose corticosteroid therapy. Hence, in the specific case of autoimmune hemolytic disorders such as AIHA, the overall effect of high-dose corticosteroid therapy needs to be specifically studied, and could possibly require the use of additional prophylactic measures, especially in patients with other underlying risk factors.

The Padua prediction score was higher in VTE + patients compared to VTE-, which aligns with the findings of Chen et al. [13]. Our analysis suggests that, despite modest discriminative ability for the Padua score (AUC of the ROC curve was 0.66), patients with the higher incidence of VTE were well identified by this score. The elevated risk was mainly due to a history of prior VTE and reduced mobility, factors that must be considered in the management of AIHA flare-ups. The Padua prediction score, despite the fact that it was originally intended for medical inpatients and not outpatients, could be an important tool for identifying high-risk patients who might benefit from early intervention, such as prophylactic anticoagulation, to prevent thrombotic events. In clinical practice, adopting such predictive models could help mitigate the risk and improve overall patient management.

ATE risk was far less studied in AIHA until now, and often mentioned anecdotally. In our cohort, patients with ATE were generally older (median age 62.7 versus 75.9 years). Age ≥ 80 years at diagnosis was associated with ATE (OR 8.93 [IC95%: 1–68], *p* = 0.022) as was the presence of ≥ 3 cardiovascular risk factors (OR 8.94 [IC95%: 1–70], *p* = 0.012). Given the increased prevalence of cardiovascular risk factors among patients who developed ATE, it is crucial to consider these factors when managing AIHA in elderly patients, especially those receiving high-dose corticosteroids, which are known to potentially worsen risk factors such as diabetes and arterial hypertension. There was no direct link between ATE and hemolytic flare-ups in the cohort. Preventive cardiovascular measures, along with anticoagulation, may be warranted to minimize the risk of arterial thrombotic events in this population.

One-year survival for all patients was 87.5%, dropping to 58% after a mean follow-up of 3.9 years. TE complications accounted for 9.4% of the deaths but infections remained the leading cause of mortality, consistent with other studies [8, 12].

Our study has some limitations, including its retrospective design and the absence of a control group. Secondly, important data such as the presence of aPLs were not available for all patients, leading to potential biases in the interpretation of these specific results. Third, we did not enter intermittent prophylactic treatments as time-varying variables, so there is a risk of immortal time bias and loss of information. Finally, due to the number of TE events we could not perform a multivariate analysis including all factors [20]. Nevertheless, this study is one of the largest in terms of cohort size for this rare disease, besides the findings regarding VTE it is the first to explore ATE in detail and propose an external validation of the Padua score on this specific population. The findings from this study could serve as a basis for future prospective trials, which could help validate these observations and develop a more standardized approach to manage the thrombotic risk in AIHA patients.

In conclusion, given the prevalence of thromboembolic complications in AIHA, it is critical that clinicians remain vigilant and proactive in managing the TE risk in AIHA. The management of AIHA patients should include VTE management considering both pharmacologic and non-pharmacologic measures to minimize patients’ risk, particularly in cases of prior splenectomy, history of VTE, bed rest due to asthenia associated with active disease, or primary wAIHA. This prevention also extends to AIHA outpatient settings, where increased vigilance is probably needed. The use of predictive models such as the Padua score could aid in identifying patients at higher risk. In elderly patients with cardiovascular risk factors, ATE prevention remains a major challenge. AIHA guidelines should include the prevention of TE in patients diagnosed with AIHA. Further prospective studies are needed to confirm these findings and improve prevention. A better understanding of the interplay between hemolysis, inflammation, and thrombosis in AIHA could ultimately lead to more effective prevention strategies, improved patient outcomes, and reduced morbidity and mortality in this population.

**Table 3:** Summary of studies on AIHA and venous arterial thrombosis

Study	Study type & follow-up	Patient number	Control	AIHA type		VTE	ATE	Splenectomy	aPLs search	Hemolysis due to TE	Other notable results
Allgood & Chaplin, 1967 [26]	Retrospective 120 months	47		wAIHA	Primary	Five PE; four caused death (10.7%)	One MI (2.1%)	Yes 5/5 VTE	-	Not specified	-
Kokori et al., 2000 [18]	Retrospective 48 months	41	41	wAIHA	Secondary (SLE)	10 TE (24%) or OR 7 (*p* = 0.07) without precising ATE or VTE		-	Yes, IgG anticardiolipin linked to AIHA	Not compared to TE	No correlation between TE & aPLs
Pullakart et al., 2002 [15]	Retrospective 82 months	30		AIHA	Primary Secondary	Eight VTE (27%); three PE (10%)	-	-	Yes 19+/30 (63%) patients; six VTE	Yes (first attack or relapse)	LCA linked to VTE (*p* = 0.03)
Hendrick et al., 2003 [14]	Retrospective 192 months	28		wAIHA cAIHA	Primary Secondary	Six VTE (21.4%); four deaths	Four (one ischemic stroke, one MI and two DIC); 14.2%	Yes three patients, no TE	Yes, zero APL out of six patients VTE	Yes	-
Bongarzoni et al., 2005 [27]	Prospective 48 months	21	42	wAIHA One cAIHA	Primary Secondary	0	Two TIA (cAIHA)	-	Yes 10+/21; 47.6%, no TE	Not compared to ATE	Hb variant rate, 5–15 g/dL Outpatient
Roumier et al., 2014 [23]	Retrospective 120 months	60		wAIHA cAIHA	Primary Secondary	12 VTE (20%)	One ischemic stroke; 1.6%	Yes, nine patients; five VTE	Yes, No link to TE	Yes (ATE/VTE)	
Barcellini et al., 2014 [12]	Retrospective 420 months	308		wAIHA cAIHA mAIHA	Primary	33 patients; 11% (11 PE, 13 DVT, five TE, one DIC, three ischemic strokes, two TIA, three MI)		Yes, more frequent TE	Yes, No link to TE	Yes (VTE, one MI related to hemolysis)	Anemia more severe linked to VTE
Lecouffe-Desprets et al., 2015 [9]	Retrospective 52 months	40		wAIHA	Primary Secondary (no cancer)	Eight patients; 20%	-	Yes, 3/8 patients	Yes, 0/8 patients	Yes	Anemia more severe linked to VTE
Chen et al., 2017 [13]	Retrospective 240 months	156	312	wAIHA cAIHA mAIHA	Primary Secondary	29%		Yes, no difference	Yes, no difference	Yes	AIHA = risk factor independent of VTE
Audia et al., 2018 [8]	Retrospective 120 months	48		wAIHA	Primary Secondary	11 patients (PE & DVT); 23%	One ischemic stroke; 2%	Yes in 2/9 TE who underwent surgery	Yes in 9/11 negative patients	Yes	
Fattizzo et al., 2022 [17]	Retrospective 51 months and prospective (12 months for 174).	287		wAIHA cAIHA	Not specified	28 patients (PE & DVT); 10%	Three MI, 2 ischemic strokes; 1,7%	Yes, 6/33 TE	Not specified	Yes	Predictors of TE: need of transfusion, RTX, CYP, infections

AIHA: autoimmune hemolytic anemia; wAIHA: warm AIHA; cAIHA: cold AIHA; mAIHA: mixed AIHA; PE: pulmonary embolism; VTE: venous thromboembolism; ATE: arterial thromboembolism; aPLs: anti-phospholipid antibody syndrome; TE: thromboembolism; MI: myocardial infarction; IgG: immunoglobulin G; OR: odds ratio; SLE: systemic lupus erythematosus; DIC: disseminated intravascular coagulation; TIA: transient ischemic attack; DVT: deep venous thrombosis; Hb: hemoglobin, RTX: rituximab, CYP: cyclophosphamide

## Electronic supplementary material

Below is the link to the electronic supplementary material.

Supplementary Material 1

## Data Availability

Data can be made available from the corresponding author on reasonable request.
