# Supplementary material for: Prediction of thrombotic events in patients with autoimmune hemolytic anemia: a multicenter retrospective observational study
Source: J Thromb Thrombolysis. 2025 Jun 22;58(7):945–54. doi: 10.1007/s11239-025-03129-8 (PMC12612021; doi:10.1007/s11239-025-03129-8)
Supplement: Supplementary file 1 — Supplementary Material 1 [file 11239_2025_3129_MOESM1_ESM.docx]

**Supplementary Table 1.** Comparison of the clinical and biological characteristics in the VTE- and VTE+ subgroups.

|  |  | Total (n=88) | AIHA VTE- (n=72) | AIHA VTE+ (n=16) | OR (CI 95%) | *p* |
| --- | --- | --- | --- | --- | --- | --- |
| Women, n (%) |  | 50 (56.8%) | 39 (54.2%) | 11 (68.8%) |  | 0.287 |
| Mean age (years) | Upon diagnosis | 63.7 | 64.1 | 61.9 |  | 0.682 |
|  | Upon inclusion | 64.9 | 64.9 | 64.6 |  | 0.948 |
| AHAI subclass | wAHAI, n (%) | 72 (81.8%) | 58 (80.6%) | 14 (87.5%) |  | 0.726 |
|  | - Primary, n (%) | 30 (41,7%) | 21 (36,2%) | 9 (64.3%) | **3.08 (0.89-11.15)** | **0.047** |
|  | - Secondary, n (%) | 42 (58.3%) | 37 (63.8%) | 5 (35.7%) |  | 0.144 |
|  | cAHAI, n (%) | 16 (18.2%) | 14 (19.4%) | 2 (12.5%) |  | 0.726 |
|  | - Primary, n (%) | 7 (53.8%) | 6 (54.5%) | 1 (50%) |  | 1 |
|  | - Secondary, n (%) | 6 (46.2%) | 5 (45.5%) | 1 (50%) |  | 1 |
|  | - Cold agglutinin disease, n (%) | 13 (81.3%) | 11 (78.6%) | 2 (100%) |  | 1 |
|  | - Post infectious disease, n (%) | 3 (18.7%) | 3 (21.4%) | 0 (0%) |  | 1 |
| Classic risks | VTE history, n (%) | 19 (21.6%) | 12 (16.7%) | 7 (43.7%) | **3.81 (1.00-14.34)** | **0.038** |
|  | Hospitalization, n (%) | 65 (73.9%) | 51 (70.8%) | 14 (87.5%) |  | 0.220 |
|  | Padua | 3.3 | 3 | 4.3 | **-** | **0.039** |
| Hemolysis | Attacks, n (%) |  |  |  |  |  |
|  | 1 | 51 (58%) | 44 (61.6%) | 7 (43.7%) |  | 0.203 |
|  | 2 | 20 (22.7%) | 16 (22.2%) | 4 (25%) |  | 0.753 |
|  | 3 | 3 (3.4%) | 2 (2.8%) | 1 (6.3%) |  | 0.457 |
|  | 4 | 8 (9.1%) | 7 (9.7%) | 1 (6.3%) |  | 1 |
|  | ≥5 | 6 (6.8%) | 3 (4.2%) | 3 (18.7%) |  | 0.071 |
|  | Biological parameters |  |  |  |  |  |
|  | Hb (g/dL) | 7.99 | 7.92 | 8.32 |  | 0.432 |
|  | LDH (UI/L)* | 1,021 | 1,048 | 868 |  | 0.604 |
|  | Total bilirubin (μmol/L)* | 40 | 40 | 40 |  | 0.729 |
|  | Free bilirubin (μmol/L)* | 34 | 36 | 27 |  | 0.283 |
| Other parameters | Splenectomy, n (%) | 9 (10.2%) | 4 (4.9%) | 5 (31.3%) | **7.50 (1.4-44.1)** | **0.009** |
|  | aPLs |  |  |  |  |  |
|  | Testing, n (%) | 32 (36.4%) | 24 (33.3%) | 8 (50%) |  | 0.210 |
|  | Positive, n (%) | 11 (34.4%) | 10 (41.7%) | 1 (12.5%) |  | 0.209 |
|  | CRP (mg/L)* | 18.8 | 16.1 | 30.6 |  | 0.093 |
| Anti-thrombotic treatment | APT, n (%) | 18 (20.5%) | 16 (22.2%) | 2 (12.5%) |  | 0.508 |
|  | DAPT, n (%) | 2 (2.3%) | 1 (1.4%) | 1 (6.25%) |  | 0.332 |
|  | Therapeutic-intensity anticoagulation, n (%) | 7 (8%) | 5 (7.9%) | 2 (12.5%) |  | 0.606 |

AIHA: autoimmune hemolytic anemia; Hb: hemoglobin; aPLs: Anti-phospholipid antibody syndrome; OR: Odds Ratio; APT: antiplatelet therapy; DAPT: dual antiplatelet therapy; LDH: lactate dehydrogenase; CRP: C-reactive protein; CI: Confidence Interval; VTE: venous thromboembolism. *: Presence of missing data.

**Supplementary Table 2.** Comparison of the clinical and biological characteristics in the ATE- and ATE+ subgroups.

|  |  | Total  (n=88) | AIHA ATE- (n=81) | AIHA ATE+ (n=7) | *p* |
| --- | --- | --- | --- | --- | --- |
| Women, n (%) |  | 50 (56.8%) | 45 (55.6%) | 5 (71.4%) | 0.694 |
| Mean age (years) | Upon diagnosis | 63.7 | 62.7 | 75.9 | 0.065 |
|  | Upon inclusion | 64.9 | 63.9 | 76.6 | 0.067 |
| Mean number of CV risk factor per patient | | 2.1 | 2 | 3.1 | 0.061 |
| Hemolysis | Biological parameters |  |  |  |  |
|  | Hb (g/dL) | 7.99 | 8.02 | 7.73 | 0.691 |
|  | LDH (UI/L)* | 1,021 | 1,043 | 757 | 0.690 |
|  | Total bilirubin (μmol/L)* | 40 | 42 | 28 | 0.330 |
|  | Free bilirubin (μmol/L)* | 34 | 35 | 25 | 0.389 |
| Other parameters | Splenectomy, n (%) | 9 (10.2%) | 4 (4.9%) | 1 (14.3%) | 0.346 |
|  | aPLs |  |  |  |  |
|  | Testing, n (%) | 32 (36.4%) | 29 (35.8%) | 3 (42.9%) | 0.702 |
|  | Positive, n (%) | 11 (34.4%) | 10 (34.5%) | 1 (33.3%) | 1 |
|  | CRP (mg/L)* | 18.8 | 19.8 | 7.8 | 0.336 |
| Anti-thrombotic parameters | APT, n (%) | 18 (20.5%) | 15 (18.5%) | 3 (42.9%) | 0.148 |
|  | DAPT, n (%) | 2 (2.3%) | 2 (2.5%) | 0 (0%) | 1 |
|  | Therapeutic-intensity anticoagulation, n (%) | 7 (8%) | 6 (7.4%) | 1 (14.3%) | 0.452 |

AIHA: autoimmune hemolytic anemia; ATE: arterial thromboembolism; CV: cardiovascular; Hb: hemoglobin; aPLs: anti-phospholipid antibody syndrome; APT: antiplatelet therapy; DAPT: dual antiplatelet therapy; LDH: lactate dehydrogenase; CRP: C-reactive protein. *: Presence of missing data.
